# The Role of Gut Microbiota and Its Metabolites in Patients with Heart Failure

**DOI:** 10.3390/biomedicines12040894

**Published:** 2024-04-18

**Authors:** Krzysztof Cienkowski, Alicja Cienkowska, Karolina Kupczynska, Agata Bielecka-Dabrowa

**Affiliations:** 1 Faculty of Medicine, Medical University of Lodz (MUL), al. Tadeusza Kosciuszki 4, 90419 Lodz, Poland; 2 Faculty of Biology and Environmental Protection, University of Lodz, ul. Gabriela Narutowicza 68, 90136 Lodz, Poland; 3 Department of Cardiology and Adult Congenital Heart Diseases, Polish Mother’s Memorial Hospital Research Institute (PMMHRI), Rzgowska 281/289, 93338 Lodz, Poland; karolinakupczynska@gmail.com (K.K.);; 4 Department of Preventive Cardiology and Lipidology, Medical University of Lodz (MUL), Rzgowska 281/289, 93338 Lodz, Poland

**Keywords:** heart failure, gut microbiota, metabolites, short-chain fatty acids (SCFAs), betaine, trimethylamine N-oxide (TMAO), phenylalanine, inflammatory markers

## Abstract

Heart failure (HF) is a significant health concern; early detection and prevention are crucial. Recent studies suggest that the gut microbiota and its metabolites may influence HF development and risk factors. We explored this relationship by examining changes in gut microbiota composition and metabolite levels in HF patients. HF patients often exhibit decreased alpha and beta diversity compared to controls, suggesting lower bacterial richness and community variation. Changes in specific bacterial phyla were observed, with decreases in Firmicutes (e.g., *Ruminococcus*) and Bacteroidetes (e.g., *Prevotella*) and increases in Proteobacteria (e.g., *Escherichia*, *Shigella*, and *Klebsiella*) and Actinobacteria. Gut-microbiota-related metabolites have been identified, potentially affecting various body systems, including the cardiovascular system. Among these are short-chain fatty acids (SCFAs), betaine, trimethylamine N-oxide (TMAO), phenylalanine, tryptophan–kynurenine, and phenylacetylgutamine (PAGIn). Although SCFAs positively affect our organisms, patients with HF have been observed to experience a decline in bacteria responsible for producing these chemical compounds. There have been indications of possible links between betaine, TMAO, phenylalanine, tryptophan–kynurenine, PAGIn, and heart failure. TMAO and phenylalanine, in particular, show promise as potential prognostic factors. However, their clinical significance has not yet been thoroughly evaluated and requires further investigation.

## 1. Introduction

Diseases related to the circulatory system are one of the leading causes of death and morbidity among people [1,2]. Heart failure (HF) represents the ultimate phase in numerous cardiac and vascular illnesses. HF is marked by the heart’s inability to effectively pump enough blood to fulfil the body’s need for nutrients and oxygen. The bleak outlook associated with it leads to elevated rates of hospitalization and mortality [3,4]. Hence, early detection and preventive measures are crucial for enhancing the outlook of individuals suffering from HF [5].

Many factors have an impact on the development of diseases in this group, including ageing, sex, hypertension, obesity, diabetes mellitus, systemic inflammation, smoking, dyslipidaemia, a sedentary lifestyle, and dietary choices [4,6].

A less obvious cause may be changes in the gut microbiota and the metabolites they produce. Numerous observational studies have indicated this correlation. Unfortunately, this relationship is only partially explained through reverse causality, limited sample sizes, and confounding factors in observational studies [5].

The human gut microbiota refers to the complex and diverse community of microorganisms that inhabit the digestive tract, primarily the large intestine. This microbiota consists of trillions of microbes, including bacteria, viruses, fungi, and other microorganisms. Most of these microbes are bacteria, crucial in maintaining human health [7,8].

The primary phyla predominating in the human gut microbiota are Bacteroidetes, Firmicutes, Actinobacteria, Proteobacteria [9], and *Cerrucomicrobia* [8]. Typically, the gut microbiota remains consistent within an individual and their family. In a healthy gut, the anaerobic groups Bacteroidetes and Firmicutes collectively account for over 90% of the total bacterial species [8]. Also abundant are *Fusobacteria*, *Verrucomicrobia* [9], *Cyanobacteria*, and *Spirochaeates* [7].

The gut microbiota is involved in essential functions, such as aiding digestion, synthesizing specific vitamins (biotin, vitamin K, etc.), and hormones, modulating the immune system, building the mucosal barrier, and protecting against harmful pathogens [5,7,10,11]. Beyond that, they also provide nutrients and energy by breaking down complex carbohydrates into simple sugars and short-chain fatty acids (SCFAs). The microbiota is responsible for the biotransformation of many chemical compounds that the human body is not adapted to carry out [7,12] (e.g., vitamin K and B group vitamins) [13]. The composition and diversity of the gut microbiota can vary significantly among individuals, influenced by genetics, diet, age, and environmental exposure [8].

The intestinal microbiota produces diverse metabolites that exhibit effects on the body, both positive and negative [14]. More research attempts to identify the relationship between these metabolites and specific diseases and even use them as potential predictive factors. Several groups of such chemical compounds that may be associated with HF have been identified. The most frequently described metabolites include trimethylamine N-oxide (TMAO), phenylalanine, tryptophan–kynurenine, phenylacetylglutamine (PAGln), and SCFAs [15].

Research on the human gut microbiota has expanded in recent years, revealing its impact on digestive health and potential connections to various systemic conditions, including immune disorders, metabolic diseases, and mental health [9]. The balance and diversity of the gut microbiota are believed to be crucial for overall well-being, and disruptions in this balance have been associated with various health issues [9,10].

Several papers claim that the gut microbiota can influence HF and risk factors through disturbances in its composition and the metabolites produced [5]. Factors that increase the risk of HF with preserved ejection fraction (HFpEF) encompass hypertension, age, gender, and obesity. New findings propose that the gut microbiota may play an independent role in influencing each risk factor, potentially through metabolites like SCFAs [6]. An adult’s microbiota is shaped by diet, previous infections, stress, hygiene practices, medication usage, and antibiotics [7].

**Figure 1:**
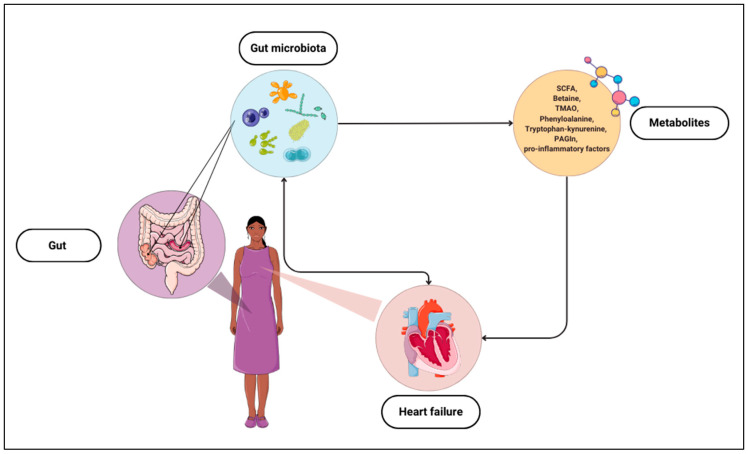
Schematic representation of the mutual influences of the gut microbiota and heart failure, including metabolites produced by microorganisms. The figure was partly generated using Servier Medical Art, provided by Servier, and licenced under a Creative Commons Attribution 3.0 Unported Licence. SCFA—short-chain fatty acids, TMAO—trimethylamine N-oxide, PAGIn—phenylacetylgutamine.

Studies on the relationship between gut microbiota, metabolites, and HF provide evidence of possible correlations and practical uses of the composition of gut bacteria or their products as markers of HF (Figure 1). Hence, it is crucial to continue research and delve into this topic to detect abnormalities related to cardiovascular diseases and potential adverse events.

## 2. Richness and Diversity of Gut Microbiota (Alpha Diversity) and Composition (Beta Diversity)

Generally, an examination of biodiversity is possible at three distinct organizational levels, which are as follows:

1. Genetic diversity—diversity of genes within a species;
2. Species diversity—diversity among the species;
3. Ecosystem diversity—diversity at ecosystem and landscape levels [16].

Additionally, Whittaker introduced three terms to assess biodiversity across spatial scales: alpha diversity, beta diversity, and gamma diversity [17]. Most studies on intestinal microbiota examine alpha and beta diversity. Alpha diversity refers to the biodiversity within a specific localised habitat or ecosystem. It measures the variety of species in a single area and provides insights into the richness and evenness of that particular environment. In other words, alpha diversity focuses on the diversity of life forms within a specific location or community [16,18]. Most commonly, it is assessed based on three indexes: Chao, Shannon, and Simpson (but not exclusively) [19].

Beta diversity is a measure of biodiversity that assesses species composition differences between habitats or ecosystems. It quantifies the degree of change or turnover in species composition. In essence, beta diversity provides insights into species diversity between distinct communities or locations, highlighting the unique species assemblages and variations in composition across different environments [16,20].

The following observations can be found in studies investigating the relationship between gut microbiota and heart defects. For a clinical evaluation of HF patients, the New York Heart Association (NYHA) scale is utilized, comprising four stages based on the symptoms and cardiovascular–respiratory capacity of the patients [21]. Zhang and co-authors, who tested congestive heart failure patients in NYHA classes III and IV and compared them with a control group, reported that the greater the degree of HF in patients, the lower the number of gut microbial species [11]. In addition, they noted that beta diversity was significantly different in all three study groups. Alpha diversity, checked using the Chao index 1, was significantly lower in NYHA class III and NYHA class IV than in the control group. Also, beta diversity was significantly different in all three groups (Table 1) [11].

Regarding alpha diversity, one study did not show differences in terms of gender, age, ethnicity/race, and patient category. However, the mentioned heterogeneity was lower with an increasing class of HF (it was also lower in patients who underwent left ventricular assist device implantation and heart transplantation patients) [22].

Similar results were obtained by Huang’s team [19]. Alpha diversity was lower in HFpEF patients than in the control group (according to the Chao index); however, there was no statistical difference in the Shannon index and Simpson index, indicating no difference in the species diversity between the two groups. In the study above, the researchers also tested beta diversity. They showed differences between patient groups, indicating changes in the gut microbiota [19].

Comparable results can be found in other studies. The composition of the gut microbiota between control and HFpEF samples differed significantly. In addition, differences in beta diversity between HFpEF and controls were independent of differences in body mass index (BMI), age, sex, hypertension, dietary score, and fibre intake. Patients with HFpEF showed significant differences from metropolitan controls in alpha Chao1 and OTU diversity measures, but there were no statistically significant differences in Shannon diversity. Furthermore, patients with HFpEF showed significant differences from metropolitan controls in alpha Chao1 and OTU diversity measures, but there were no statistically significant differences in the Shannon index [6].

Another study has shown significant differences between the two types of microbiota diversity [23]. The alpha diversity metrics, including Chao1, PD whole tree, and Shannon indices, exhibited a significant decrease in severe chronic heart failure (CHF) compared to the control group, alongside a noteworthy disparity in beta diversity between the two groups. When it comes to the Chao1 index, Wang et al. obtained analogous results [24].

Peng et al. analysed the gut microbiota among small cohorts, specifically examining faecal samples of 33 HF patients without sarcopenia (HF), 29 HF patients with sarcopenia (SHF), and 15 controls [25]. Significant differences were observed among the control, HF, and SHF groups regarding diversity (observed species index and Chao1) and abundance (Simpson and Shannon indexes). These indices showed similarities between the HF group and the SHF group. Beta diversity indicates the diversity between habitats in microbial community structure, evaluated using Bray and Curtis distances. Significant separations were found between the control and HF or SHF groups. However, no significant variations were observed in the microbial community compositions between the HF and SHF groups. The researchers’ results suggest the dysbiosis of the gut microbiota in the HF and SHF groups compared to the control group [25].

Other results were obtained by Drapkina and his team [14]. They examined the composition of the gut microbiota in three groups of patients: those with atherosclerotic cardiovascular disease, HF patients with reduced ejection fraction (HFrEF), and HFpEF patients. The data obtained were compared with the microbiota of people without these diseases (the control group). Six alpha diversity indexes were used to assess the samples’ richness and evenness estimates. No notable distinctions were noted among the patient groups, except for the Faith index, which displayed a significant difference between the HFrEF and control groups [14]. Hayashi and co-authors also noted the lack of differences in alpha diversity (according to the Shannon diversity index) between the patient and control groups [26]. In contrast, although there were no variations in alpha diversity between the groups, the arrangement based on beta diversity metrics revealed the segregation of groups across various components of variation [15].

It was also suggested that the gut microbiota’s alpha and beta diversity differs significantly between control and HF patients. Typically, the microbiota diversity is lower in sick patients. Interestingly, the diversity mentioned was not dependent on personal characteristics, e.g., age, gender, and BMI [23].

**Table 1:** Comparison of alpha and beta diversity, using statistical indexes, between groups of patients with heart failure (HF) and controls (GCs). If the result in the table indicates “HF < CG”, the result was statistically significant.

Study	Alpha Diversity	Beta Diversity
Zhang et al. [11]	HF < GC	significantly different in all groups
Huang et al. [19]	HF < GC	significantly different in all groups
Beale et al. [6]	HF < GC	significantly different in all groups
Sun et al. [23]	HF < GC	significantly different in all groups
Wang et al. [24]	HF < GC	significantly different in all groups
Peng et al. [25]	HF < GC	significantly different in all groups
Drapkina et al. [14]	no significant difference in all groups	has not been studied
Hayashi et al. [26]	no significant difference in all groups	has not been studied
Kilic et al. [15]	no significant difference in all groups	significantly different in all groups

Study

Alpha Diversity

Beta Diversity

Significant differences in the diversity and abundance of gut microorganisms observed between groups indicate the complexity of macrobiotic changes in heart diseases and accompanying sarcopenia. Despite the lack of significant differences between HF and SHF groups in microbiota composition, existing distinctions between the control group and groups of patients with HF and/or sarcopenia may have significant health implications. Further research on mechanisms related to gut microbiota dysbiosis in the context of HF and potential therapies aimed at restoring macrobiotic homeostasis is essential for better understanding these processes and developing new therapeutic strategies.

## 3. Changes in the Composition of the Gut Microbiota

The gut microbiota primarily consists of microorganisms belonging to the Firmicutes, Bacteroidetes, Actinobacteria, and Proteobacteria phyla [9]. The most significant changes are observed within these phyla. In recent years, researchers have increasingly addressed the topic of the association between gut microbiota and HF. Most studies indicate a decrease in the abundance of Firmicutes [6,11,23,25] and Bacteroidetes [11,23,25] bacteria in patients with HF. However, in one study conducted by Yuzefpolskaya, an increase in Bacteroidetes was observed [22].

It should be mentioned that within the Firmicutes phyla, there are bacteria such as *Clostridium*, *Ruminococcus*, *Butyricicoccus*, *Eubacterium*, *Faecalibacterium*, *Blautia*, *Lachnospira*, *Megamonas*, *Agathobacter*, *Ruminiclostridium*, *Lactobacillus*, *Enterococcus*, *Staphylococcus*, *Streptococcus*, and *Veillonella*, *Roseburia* [27,28].

Some research studies have observed a decrease in bacteria from the *Ruminococcus* and *Butyricicoccus* genera (Table 2). This is significant because these bacteria produce essential metabolites, including SCFAs, which have a beneficial impact on the host organism [6,11,19,29].

In contrast to the general trend of decreased *Ruminococcus* genus abundance, several studies have noted an increase in a specific species, namely *Ruminococcus gnavus* [30,31]. Interestingly, its elevated level has also been observed in conditions such as inflammatory bowel disease and metabolic disorders like obesity, type 2 diabetes, and non-alcoholic fatty liver disease. However, knowledge about this species and its potential associations with various conditions remains limited [32].

A decrease in bacteria from the *Eubacterium* and *Faecalibacterium* genera has also been observed in patients with HF. The reduction in species like *Eubacterium rectale* and *Faecalibacterium prausnitzii* is particularly significant, as these microorganisms also produce SCFAs, specifically butyrate. This metabolite influences the functioning of the gut barrier and exhibits anti-inflammatory effects [33,34].

The level of *Blautia* was also reduced, and there are suspicions of its potential anti-inflammatory properties [29].

In addition to the mentioned genera, a decrease in other Firmicutes genera, such as Lachnospira, Megamonas, Agathobacter, and Ruminiclostridium, was noted [11,19,26].

Despite an overall decrease in bacteria from the Firmicutes phylum, some genera within this phylum experienced growth in patients with HF. Several studies reported increased genera such as *Lactobacillus*, *Enterococcus*, *Streptococcus*, *Veillonella*, and *Roseburium* [11,19,23,25,30,31].

A decrease in Bacteroidetes, a phylum of bacteria, was observed in patients with HF (except for one study). This phylum includes genera like *Bacteroides*, *Prevotella*, *Parabacteroides*, and *Alistipes*. Among them, a significant decrease in *Prevotella* was observed, and these microorganisms were found to play an important role in the metabolism of essential amino acids in the intestines [33]. On the other hand, an increase in bacteria classified into the genera *Alistipes* and *Parabacteroides* was noted [11].

At this point, it is worth noting the Firmicutes-to-Bacteroidetes ratio, which is commonly utilized as an indicator of gut dysbiosis [35], and its relevance in cardiovascular diseases has also been examined. Emoto et al. observed a heightened Firmicutes/Bacteroidetes ratio among coronary artery disease patients compared to the control cohort. Furthermore, deviations in the Firmicutes/Bacteroidetes ratio were linked to risk factors for cardiovascular diseases such as type 2 diabetes, dyslipidemia, and hypertension [36]. The precise significance of this metric in HF remains uncertain due to a scarcity of studies investigating this correlation. While one study documented a decline in the Firmicutes/Bacteroidetes ratio in HF patients, it failed to reach statistical significance [6]. Similarly, another study did not detect significant variances in this indicator [26]. To gain deeper insights into the significance of this indicator in HF patients, further research conducted on larger patient cohorts is imperative.

An increase was observed in the subsequent phyla types of bacteria, including Actinobacteria, Proteobacteria, and *Synergistetes* [6,11,19,22,23,25,26]. Proteobacteria mainly consist of Gram-negative bacteria with lipopolysaccharides (LPSs) on their outer membrane. LPSs have a strong immunogenic effect and exhibit various detrimental actions in the host’s body, causing endotoxemia and inflammation [37]. Many pathogenic genera of bacteria belong to this phylum, such as *Escherichia*, *Shigella*, and *Klebsiella*. Many of these bacteria are associated with adverse effects, and the growth of all three phyla has been observed in the gut microbiota of patients with HF. Other studies noted increased pathogenic microorganisms, including *Campylobacter*, *Salmonella*, *Yersinia*, and *Candida* fungi [38] and *Haemophilus* [30]. None of the studies presented the impact of several microorganisms on the risk of certain cardiovascular diseases. We showed that an increase in *Shigella* by every one unit increased the risk of myocarditis by 38.1% and hypertrophic cardiomyopathy by 13.3%. In comparison, elevated levels of *Candida* by one unit were associated with an increased risk of chronic kidney disease by 7.1%. There was no evidence that *Candida, Shigella*, and *Campylobacter* were associated with an increased risk of HF.

It was also proposed that individual bacteria might not individually impact the initiation of HF, and the collective function of the gut microbiota may play a more significant role in influencing the risk of HF [5].

Another genus of bacteria belonging to Proteobacteria is *Sutterella*, but unlike the previous examples, a decrease in this group was recorded [19].

Within the Actinobacteria phylum are bacteria from the *Bifidobacterium* genus, which showed a significant increase in the microbiota of patients with HF [11,25,26].

However, Modrego et al. presented an increase in *Bifidobacterium* levels after 12 months of appropriate HF treatment in their study. Furthermore, they also demonstrated the positive impact of this phylum of bacteria. They showed that higher levels of *Bifidobacterium* were associated with lower levels of pro-inflammatory markers and N-terminal pro-B-type natriuretic peptides (NTpro-BNP) and were positively correlated with markers of endothelial function and improved intestinal barrier function [39].

The next genus within the Actinobacteria phylum is *Collinsella*. Interestingly, the growth of *Collinsella* was observed in patients with atherosclerosis or type 2 diabetes mellitus. However, in patients with HF, this genus was decreased. A lower level was also found in patients with HF and coexisting ischemic heart disease or diabetes, leading to the conclusion that the *Collinsella* genus may be specific to HF [29].

**Table 2:** Changes in bacterial composition at the phylum, genus, and species levels. Explanation: ↑ indicates an increase, ↓ indicates an decrease.

Phylum	Changes	Study	Genus	Changes	Study
Proteobacteria	↑	[6,11,22,23,25]	*Escherichia*	↑	[11,23,30]
			*Shigella*	↑	[11,23,25,30,38]
			*Salmonella*	↑	[38]
			*Klebsiella*	↑	[11]
			*Campylobacter*	↑	[38]
			*Yersinia*	↑	[38]
			*Suterella*	↓	[19]
Actinobacteria	↑	[23,25,26]	*Bifidobacterium*	↑	[11,25,26]
				↓	[39]
			*Collinsella*	↓	[29]
Firmicutes	↓	[6,11,23,25]	*Lactobacillus*	↑	[11,19,23,25,30]
			*Enterococcus*	↑	[19,23,25]
			*Streptococcus*	↑	[6,11]
			*Veillonella*	↑	[40]
			*Roseburium*	↑	[11]
			*Megamonas*	↑	[11]
				↓	[19,26]
			*Ruminococcus*	↓	[19,26]
			*Butyricicoccus*	↓	[19]
			*Eubacterium*	↓	[33,41]
			*Faecalibacterium*	↓	[23,29]
			*Blautia*	↓	[29]
			*Lachnospira*	↓	[19]
			*Agathobacter*	↓	[11]
			*Ruminiclostridium*	↓	[19]
Bacteroidetes	↑	[22]	*Alistipes*	↑	[11]
			*Parabacteroides*	↑	[11]
	↓	[11,23,25]	*Prevotella*	↓	[33]
Synergistetes	↑	[19]	-	-	-
**Phylum**	**Species**		**Changes**	**Studies**	
Firmicutes	*Ruminococcus gnavus*		↑	[30,31]	
	*Eubacterium Rectale*		↓	[33,34]	
	*Faecalibacterium prausnitzii*		↓	[33,34]	

## 4. Selected Metabolites and Their Correlations with Specific Pathological Conditions

The intestinal microbiota generates many metabolites exhibiting significant biological activities in the human organism. These activities encompass both advantageous and unfavourable effects. An expanding body of scientific endeavours to establish the correlations between distinct metabolites and specific pathological conditions, with the prospect of employing them as prognostic indicators.

### 4.1. Short-Chain Fatty Acids (SCFAs)

SCFAs are metabolites formed by the intestinal microbiota through the fermentation of carbohydrates that humans do not assimilate. They contain up to six carbon atoms in their chain, including acetic acid, propionate, butyric acid, valeric acid, caproic acid, and acrylic acid [42].

SCFAs have many vital functions in the human body. They are used as an energy source by intestinal epithelial cells and influence the integrity of the intestinal barrier. In addition, they are linked to the production of intestinal hormones and the stimulation of water and sodium absorption, and, perhaps most importantly, in the context of the development of specific disease entities, they have anti-inflammatory effects [43,44].

It has been observed that SCFAs decrease the secretion of proinflammatory cytokines, like interleukin-6 (IL-6) and interleukin-8 (IL-8), and inhibit the expression of intercellular adhesion molecule-1 (ICAM-1) and vascular cell adhesion molecule-1 (VCAM-1) [45]. In addition, it has been theorised that SCFAs can lower blood pressure, and their supplementation prevents elevated blood pressure [46,47,48,49,50,51].

Moreover, SCFAs have beneficial effects on the heart because they improve myocardial repair, maintain normal heart contractile function, and maintain electrical stability [6,52].

In studies with patients, it has been observed that the gut microbiota in HF sufferers differs significantly from control groups. As mentioned, there was generally a decrease in the bacteria responsible for SCFAs production, such as *Ruminococcus* [6,11], *Butyricicoccus* [19], *Eubacterium* (*E. rectale*), and *Faecalibacterium* (*F. prausnitzii*), and this may in turn translate into a decrease in SCFAs levels in the bloodstream [31,34]. A study by Vlasov et al. may confirm this. They observed that patients with chronic HF had lower levels of *Ruminococcus*-specific fatty acids than the control group [41].

The association of the abundance of SCFAs-producing bacteria and the levels of these acids with HF may also be suggested by the results provided by Modrego and co-workers. They observed that after 12 months of treatment of newly diagnosed HF, SCFAs concentrations changed significantly. Faecal acetate, propionate, and butyrate concentrations were significantly higher after 12 months of follow-up (both faecal acetate and butyrate concentrations were already significantly higher after six months). An increase in SCFAs-producing bacteria was also observed after the indicated treatment time. They also found a link that butyrate concentrations in faecal samples were negatively correlated with NT-proBNP and pro-inflammatory factor (ICAM-1) levels and positively correlated with anti-inflammatory monocytes and endothelial progenitor cells. A negative correlation was also observed between acetate and NT-proBNP, and a positive correlation was observed with left ventricular ejection fraction (LVEF) and endothelial progenitor cells. Propionate correlated positively with anti-inflammatory activity [39].

An investigation of the relationship between SCFAs levels and the risk of cardiovascular disease was also attempted. In his study, Luo observed that as propionic acid increased by every one unit, it was associated with a lower risk of HF by 2.0%. In contrast, it increased the risk of myocardial infarction (MI) by 2.8% [5].

The utility of acetate in predicting adverse cardiovascular events was also tested. Based on the PROSPER 13 cohort study, it was determined to be inversely correlated with HF hospitalization. In contrast, the results of the second cohort study, FINRISK 1997, did not confirm this. Therefore, further studies are needed to determine whether the mentioned compound has the potential to be used as a prognostic indicator [53].

### 4.2. Betaine and Trimethylamine N-oxide (TMAO)

One of the metabolites produced by the gut microbiota is phosphatidylcholine derivatives. These compounds are chemically related and may have significant associations with cardiovascular diseases. Phosphatidylcholine supplied through diet can further transform choline and betaine, which are then metabolised into trimethylamine (TMA) with the assistance of the gut microbiota. Subsequently, TMA enters the bloodstream and is transported to the liver and then oxidised into trimethylamine N-oxide (TMAO) [54,55].

Luo et al. demonstrated that an increase in betaine by every one unit is associated with a 1.4% increased risk of HF and a 1.7% increased risk of heart attack, while also correlating with a 3.7% decreased risk of chronic kidney disease [5]. The research team highlighted that betaine may directly contribute to an elevated risk of HF and indirectly influence it by increasing the likelihood of MI, which in turn could affect cardiac function. These findings align with previous reports regarding the correlation between betaine levels, HF, and MI [5]. Moreover, a study by Lever et al. reported that serum betaine concentrations were independently associated with the risk of HF [56,57]. Furthermore, Tang et al. noted that increased levels of betaine were linked to a decline in diastolic function among patients already diagnosed with HF [54]. Additionally, another study revealed a connection between betaine levels and the frequency of MI [24].

However, no direct association was established between betaine and biomarkers indicating inflammatory responses or endothelial damage. This has led to speculation that betaine might influence the risk of HF through mechanisms other than inflammation and endothelial damage [54].

Other studies report even the anti-inflammatory and antioxidant effects of betaine, which are believed to have favourable effects on preventing liver, cardiovascular, and neurodegenerative diseases [58]. These properties may be due to betaine’s influence on homocysteine metabolism, reducing its levels and mitigating potential adverse effects [51,58,59].

There is a growing interest within the scientific community regarding TMAO, with studies exploring its connections to various disease conditions, notably those affecting the cardiovascular system and kidneys. Furthermore, more research efforts are directed towards examining its potential clinical applications.

In their study, Romano et al. aimed to identify bacteria involved in TMAO production, identifying nine strains primarily from the Firmicutes and Proteobacteria phyla [60]. These observations suggest that the gut microbiota may influence circulating TMAO levels by regulating intestinal TMA synthesis [55].

Some sources revealed that an incremental rise in TMAO concentrations by each one unit is correlated with a 7.1% elevation in the likelihood of elevated systolic blood pressure [5], consistent with findings from a prior meta-analysis by Ge et al. [61].

Brun et al. presented a plausible mechanism for this correlation, suggesting that it may involve TMAO’s ability to inhibit endothelial nitric oxide synthases (eNOSs) and induce oxidative stress, ultimately leading to endothelial cell dysfunction [62]. Moreover, an increased TMAO level was linked to a 3.1% rise in the risk of chronic nephritis and a 1.6% increase in the risk of type 2 diabetes mellitus [5].

Increased TMAO, choline, and betaine levels were linked to elevated serum levels of NT-proBNP and more pronounced left ventricular diastolic dysfunction without affecting systolic dysfunction or biomarkers indicative of inflammation and endothelial function [54].

There were statistically significant correlations between TMAO, choline, and betaine levels and various echocardiographic parameters. Tang et al. observed consistent positive associations between TMAO and indicators of diastolic dysfunction, particularly with E mitral/Ea septal and the left atrium volume index. Conversely, no significant correlations were detected between TMAO, choline, betaine levels, and LVEF or left ventricle dimensions [54].

In summary, elevated serum levels of TMAO, choline, and betaine are associated with more advanced left ventricular diastolic dysfunction and predict poorer long-term adverse clinical outcomes in chronic systolic HF. However, only higher TMAO levels in serum were associated with poor prognosis after adjustment for cardiorenal markers [54].

Due to TMAO’s potential association with cardiovascular disease, studies have been conducted to assess its predictive value. The aim is to use changes in TMAO concentrations as a prognostic marker of HF as an independent tool to predict adverse events in HF patients.

Suzuki et al. conducted the initial assessment of TMAO in acute HF and discovered its role as a predictor for endpoint composed of death and death/HF within one year. Nevertheless, upon adjustment for renal function parameters, TMAO’s independent predictive capability was lost, likely due to notable associations between TMAO and renal function parameters [63].

Integrating TMAO with the existing clinical risk assessment algorithm enhanced the stratification of in-hospital mortality risk, and incorporating NT-proBNP into this model further improved the prediction of death/HF within one year. Furthermore, it was noted that patients with elevated levels of both markers (TMAO and NT-proBNP) exhibited the highest risk of death/HF [55].

Zhang et al. observed that patients diagnosed with congestive HF displayed notably elevated TMAO levels, which were associated with a 3.4-fold higher risk of mortality. Moreover, increased TMAO levels indicated a heightened mortality risk over five years [55].

Likewise, Tang et al. correlated elevated levels of TMAO, along with choline and betaine, with an increased risk of adverse clinical events, such as death or the requirements for transplantation, over a 5-year timeframe. However, only higher TMAO levels independently predicted adverse clinical events, regardless of age, eGFR (estimated glomerular filtration rate), mitral E/septal Ea, and NT-proBNP levels [54,64].

It is worth noting that the predictive value of TMAO varied among different ethnic groups. Elevated TMAO levels were significantly linked to adverse outcomes solely among patients of Caucasian descent, while they held less relevance among patients of other ethnicities [65]. Moreover, even after accounting for interfering factors, TMAO levels exhibited variations based on geographic regions, and their relationships with HF outcomes also varied [66].

It has also not been clearly defined whether TMAO has predictive value only for HFrEF or whether it may also be helpful in HF patients with reduced HFpEF.

One clinical study revealed that elevated TMAO levels were predictive of cardiovascular events, specifically in individuals with HFrEF, but lacked significant prognostic value in those with HFpEF [67].

However, another investigation focusing on HFpEF found that TMAO levels contributed to risk assessment in HFpEF patients, particularly when NT-proBNP levels were not elevated. Moreover, the author suggested combining NT-proBNP and TMAO concentrations could offer more informative prognostic insights for individuals with HFpEF [68].

### 4.3. Phenylalanine

Phenylalanine is another metabolite that could impact the circulatory system. According to Luo et al.’s research, increases of 1.7%, 8.0%, and 2.0% in phenylalanine by each unit was associated with a higher risk of developing HF, hypertrophic cardiomyopathy, and valvular heart disease, respectively [5].

Various studies have reported similar results, indicating significantly elevated phenylalanine concentrations in HF patients compared to control groups [69,70,71,72,73].

Due to its association with HF, the prognostic value of phenylalanine was evaluated. Delles and colleagues conducted a study in this regard, utilizing metabolomics to assess two cohort studies, PROSPER 13 and FINRISK 1997. Their findings showed that phenylalanine levels are elevated and independently associated with incident heart failure hospitalization. However, incorporating phenylalanine into the predictive model did not enhance HF prediction beyond established clinical prognostic factors and NT-proBNP levels, indicating its likely limited practical utility in clinical settings [53].

In their study on the predictive value of phenylalanine, Chen et al. even identified a threshold level set at 112 μM, which was associated with poorer outcomes. Patients with phenylalanine concentrations ≥ 112 μM exhibited markedly higher mortality rates compared to those with concentrations below 112 μM (80.5% vs. 24.3%). Moreover, Kaplan–Meier analysis revealed that phenylalanine levels surpassing 112 μM were linked to a diminished cumulative survival rate [74].

Furthermore, elevated phenylalanine levels were linked to higher scores on the APACHE II and SOFA scales, increased C-reactive protein levels, and the need for inotropic agents, alongside cytokine alterations suggesting immunosuppression and malnutrition. Meanwhile, lower levels were associated with reduced prealbumin and transferrin levels [74].

Moreover, subsequent multifactorial analysis demonstrated that phenylalanine levels ≥ 112 μM independently predicted one-year mortality, regardless of age, APACHE II and SOFA scores, atrial fibrillation, as well as C-reactive protein, cholesterol, prealbumin, transferrin, interleukin-8, and interleukin-10 levels [74].

### 4.4. Tryptophan–Kynurenine Pathway

Metabolites within the tryptophan–kynurenine pathway warrant examination due to their potential correlation with cardiovascular ailments. In their investigation, Luo et al. found that elevations in tryptophan levels per unit were associated with increased relative risks of HF and elevated systolic and diastolic blood pressure (2.1%, 14.8%, and 6.9%, respectively). Conversely, the relative risks of hypertrophic and dilated cardiomyopathy decreased by 19.2% and 20.2%, respectively. Furthermore, each unit increase in kynurenine levels was linked to a 3.0% decrease in the relative risk of chronic kidney disease [5].

Previous research has also highlighted the connection between tryptophan and HF. Tang and colleagues utilized metabolomics to reveal a strong association between tryptophan levels and the onset of HF [75].

Similarly, Razaquin et al. found a comparable correlation in their study, indicating that higher baseline kynurenine-to-tryptophan ratio levels and elevated levels of kynurenine, kynurenic acid, and quinolinic acid were associated with an increased risk of HF [76]. Additionally, specific metabolites within this group indicated the clinical severity of HF. Specifically, quinolinic acid and the kynurenine-to-tryptophan ratio were linked to decreased exercise tolerance, elevated levels of pro-inflammatory markers, and an amplified mortality risk [77].

Elevated levels of metabolites in the kynurenine pathway have been implicated in various other cardiovascular conditions. Specifically, an increased ratio of kynurenine to tryptophan has been linked to a heightened risk of acute coronary events and cardiovascular mortality [78,79]. Furthermore, kynurenic acid has shown a significant association with a composite outcome comprising stroke, myocardial infarction, and cardiovascular mortality [80]. Besides HF, quinolinic acid has also been associated with atrial fibrillation [76].

### 4.5. Phenylacetylgutamine (PAGIn)

Increasing attention in the context of cardiovascular diseases is also being directed towards a gut-microbiota-dependent metabolite known as phenylacetylglutamine (PAGln). It was demonstrated that the concentration of PAGln correlated with the presence of HF and indicators of its severity, such as reduced LVEF and elevated NTproBN levels. This association remained significant even after accounting for traditional risk factors and kidney function [81,82].

Individuals diagnosed with HF exhibited higher systemic PAGln levels compared to those without HF. Moreover, PAGln concentration was found to be linked to HF risk, irrespective of coronary artery disease status and across various HF phenotypes [81].

Moreover, individuals exhibiting normal left ventricular systolic function and kidney function displayed associations between PAGln concentrations and cardiovascular disease, LVEF, and NT-proBNP. This suggests that the correlation between PAGln and these phenotypes manifests even before the clinically apparent onset of HF or the development of comorbidities like kidney dysfunction. Additionally, the observation that PAGln levels mirror NT-proBNP levels in individuals with preserved left ventricular ejection fraction hints at the potential involvement of PAGln, and consequently gut microbiota, in the progression of HFpEF [81]. Beyond HF, PAGln has previously been linked to cardiovascular disease and the occurrence of serious adverse cardiovascular events (myocardial infarction, stroke, or death) [83].

Despite the plethora of findings discussed, the overall impact of PAGln on HF development remains ambiguous. Animal studies have revealed that PAGln induces a negative inotropic effect under sympathetic stimulation, potentially contributing to decreased left ventricle systolic function and EF [84,85]. However, it could be argued that this effect might benefit HF patients, akin to beta-adrenergic therapy [81]. It is also unclear whether PAGln-induced NT-proBNP production represents an adaptive or maladaptive process in HF.

## 5. Endotoxemia and Inflammatory Markers

There are speculations that some gut microbiota metabolites may intensify inflammation and contribute to an increased risk of HF. Some studies report that patients with HF exhibit a different profile of inflammatory markers compared to the general population, and concentrations of inflammatory indicators also vary depending on the stage of HF.

In a study conducted by Vlasov et al., a direct correlation was observed at medium power between C-reactive protein (CRP) and IL-6 concentrations and key parameters of clinical severity. The strongest correlations with pro-inflammatory factors were noted for NT-proBNP, chronic HF stages, and the intensity of oedema. Additionally, a moderate direct correlation was found between CRP levels and the duration of patients’ worsening conditions [41].

Yuzefpolskaya also observed differences in the concentrations of pro-inflammatory factors depending on the stage of HF. Inflammatory state and oxidative stress were elevated in patients with NYHA class IV HF compared to other patients with NYHA class I-III. All inflammatory biomarkers increased with the progression of HF from class I to IV. Still, they were lower among patients treated with a left ventricular assist device (LVAD) or patients who received a heart transplant (HT) compared to class IV patients. Patients with NYHA class IV HF also exhibited decreased gut microbiota diversity and increased endotoxemia, an enhanced inflammatory state, and increased oxidative stress. However, it was observed that the inflammatory state and oxidative stress levels were lower in patients treated with LVAD or patients who received a heart transplant compared to NYHA class IV patients, while decreased gut diversity and endotoxemia persisted in patients with LVAD and HT [22].

However, data regarding endotoxemia in HF patients are not conclusive, as different results were presented in another study. Vlasov et al. observed that CHF patients exhibited a lower median LPS levels than the control group. They also noted the highest indicators of endotoxemia, Gram-negative bacteria, cocci, rods, and fungi in HF groups among patients with NT-proBNP levels ranging from 400 to 2000 pg/mL [41]. It is worth taking a closer look at the issue of endotoxemia because disturbances in the balance of gut bacteria can lead to an increase in the permeability of the intestines, allowing more bacteria, their byproducts, and LPS to pass into the bloodstream [86,87,88]. Previous research has shown that these disruptions in gut bacteria and the resulting rise in endotoxemia levels, along with markers of inflammation and oxidative stress, contribute to the development of non-alcoholic fatty liver disease (NAFLD) and non-alcoholic steatohepatitis [89]. Similarly, elevated oxidative stress levels are observed in cardiovascular diseases, potentially linking them to a higher risk of developing cardiovascular issues among patients with NAFLD [90]. These findings suggest a need to explore the relationship between endotoxemia and oxidative stress further, for instance, by using markers like serum sp-NOX2 and urinary 8-iso-PGF2 alpha, particularly in patients with cardiovascular conditions such as heart failure.

Studies conducted by Modrego and colleagues demonstrated that appropriate treatment can lower the levels of pro-inflammatory factors in patients with HF. After 12 months of administering suitable medications to patients with newly diagnosed HF, the profile of inflammatory mediators changed.

After this treatment period, levels of pro-inflammatory factors such as ICAM-1, IL-6, IL-18, CRP, sCD163, tumor necrosis factor alfa (TNF-α), and VCAM-1 were significantly reduced. Meanwhile, the levels of classical non-activated monocytes and circulating endothelial progenitor cells, which may be responsible for replacing dysfunctional endothelium, significantly increased [39].

It is also worth noting that another study did not show any statistically significant differences between the control group and patients with HF. In the study by Kilic et al., the Kruskal–Wallis test indicated no statistically significant differences between HF patients and control groups for Gal-3, IL-1, IL-6, CRP, endotoxin, LBP, or TNF. Kendall’s rank correlation coefficient also did not demonstrate a statistically significant association between levels of inflammatory markers and the richness and diversity of microbiota (at the phylum level) [15].

## 6. Gut Microbiota Interactions with Cardiovascular Drugs

Considering the potential interactions between the gut microbiota and prescribed medications is essential. Research indicates that over 24% of non-antibiotic drugs can impact gut microbiota, changing their environment and metabolic byproducts [91]. Additionally, it is essential to recognize that this relationship can work both ways: gut microbiota can influence drug metabolism and some drugs can affect gut microbiota.

Many drugs used in cardiology, such as angiotensin-converting enzyme inhibitors (ACEIs) in HF treatment, target the renin–angiotensin–aldosterone (RAA) system, and metabolites produced by gut microbiota can influence this system. SCFAs (butyrate, acetate, or propionate) regulate the RAA system by inhibiting angiotensin II, thus decreasing blood pressure. However, as described in our work, a reduction in bacteria-producing SCFAs can be observed in HF, which may be associated with adverse effects on the circulatory system [92,93]. Nonetheless, some metabolites have opposing effects. Succinate, an intermediate metabolite of propionate synthesis, increases RAA system activity via the GPR91 receptor in the kidneys under high glucose concentrations [94]. It has also been observed that TMAO can prolong the blood-pressure-raising effect caused by angiotensin II [94]. Additionally, the long-term administration of TMAO reduces angiotensin II type 1 receptor activity but increases angiotensin II type 2 receptor activity [95,96]. Interestingly, angiotensin II can also interact with the gut microbiota and cause an increase in the Firmicutes/Bacteroidetes ratio (a dysbiosis indicator) [97]. On the other hand, ACEIs have a beneficial effect on the gut microbiota, modifying intestinal barrier permeability and thereby reducing TMA leakage into the bloodstream [98].

There is speculation about an interaction between gut microbiota and beta-adrenergic drugs, as the adrenergic system, including the intestines, is present throughout the body. It is suspected that the long-term use of metoprolol and atenolol may affect the composition of the gut microbiota, leading to dysbiosis and favouring arterial hypertension [99]. However, nebivolol has low intestinal permeability, which affects its low bioavailability [100]. It has also been observed that bisoprolol, nadolol, pindolol, and talinolol have slow distribution in a toxic environment, and it is suspected that gut dysbiosis may also create unfavourable conditions for these drugs [101].

There are reports on the beneficial effects of sodium–glucose co-transporter-2 (SGLT2) inhibitors on gut microbiota. Studies on mice have shown that luseogliflozin increases SCFAs production [102]. It has also been shown that SGLT2 inhibitors have a protective effect on the intestinal mucosa, reducing the influx of inflammatory metabolites associated with gut microbiota, such as lipopolysaccharides and endotoxins [102,103].

Some bacteria can also metabolize drugs into their inactive form, as with digoxin, which in 10% of patients is transformed by *Eggerthella lenta* bacteria, consequently reducing the drug’s efficacy [104]. Additionally, a diet rich in arginine weakens the effect of digoxin by affecting its fluctuation in the blood [105].

It is suspected that the gut microbiota may also influence the popular cardiology drug aspirin by altering its bioavailability; however, a reciprocal interaction has also been observed, as aspirin use may impact changes in gut microbiota composition [27,106]. The gut microbiota also affects the metabolism and reduces the bioavailability of amlodipine [107].

There is speculation that drug–microbiota interactions may also occur with statins. Differences in microbiota composition have been observed in patients who use statins compared to non-users [108]. It is also worth noting that statins may cause increased gut permeability, contributing to inflammation and adverse effects within the intestines, especially at the neuromuscular junction [109].

However, drug–microbiota interactions are still poorly understood, partly due to numerous confounding factors and variables such as comorbidities, polypharmacotherapy, genetic variability, diet, lifestyle, etc., significantly hindering conclusive results.

## 7. Conclusions

Evidence suggests a potential link between imbalances in the gut microbiota, known as dysbiosis, and HF. Investigations into alterations in bacterial composition and metabolite levels hold promise for developing new diagnostic and risk assessment methods for HF. However, research teams often include other types and categories of heart failure, e.g., NYHA category, HFrEF, and HFpEF, which significantly hinders the overall discussion and comparison of findings on the microbiota–heart failure relationship.

Further research is needed to grasp these intricacies and establish clear cause-and-effect connections comprehensively.

## Data Availability

Not applicable.
